# Molecular Basis and Clinical Features of Neuroblastoma

**DOI:** 10.31662/jmaj.2021-0077

**Published:** 2021-09-01

**Authors:** Junko Takita

**Affiliations:** 1 Department of Pediatrics, Graduate School of Medicine, Kyoto University, Kyoto, Japan

**Keywords:** neuroblastoma, anaplastic lymphoma kinase (ALK), MYCN, chromosomal copy number alterations, spontaneous regression

## Abstract

Neuroblastoma, a neoplasm of the sympathetic nervous system, originates from neuroblastoma stem cells during embryogenesis. It exhibits unique clinical features including a tendency for spontaneous regression of tumors in infants and a high frequency of metastatic disease at diagnosis in patients aged over 18 months. Genetic risk factors and epigenetic dysregulation also play a significant role in the development of neuroblastoma. Over the past decade, our understanding of this disease has advanced considerably. This has included the identification of chromosomal copy number aberrations specific to neuroblastoma development, risk groups, and disease stage. However, high-risk neuroblastoma remains a therapeutic challenge for pediatric oncologists. New therapeutic approaches have been developed, either as alternatives to conventional chemotherapy or in combination, to overcome the dismal prognosis. Particularly promising strategies are targeted therapies that directly affect cancer cells or cancer stem cells while exhibiting minimal effect on healthy cells. This review summarizes our understanding of neuroblastoma biology and prognostic features and focuses on novel therapeutic strategies for this intractable disease.

## Introduction

Neuroblastoma is the most common childhood solid tumor. It arises from embryonal neural crest tissue and accounts for approximately 15% of all pediatric oncology deaths. The prevalence is 10.7 cases per 1,000,000 persons aged 0-14 years and there are approximately 800 new cases of neuroblastoma per year in the United States and occurs in 150-200 children each year in Japan^(1), (2), (3)^. The median age at presentation is 23 months and less than 10% of the cases are diagnosed after the age of 5 years ^(1), (2)^. This disease has a remarkable variation in clinical features, ranging from a localized disease with spontaneous regression to aggressive progression despite intensive treatment ^(2)^. Although most tumors are sporadic, neuroblastoma rarely occurs as a familial or syndromic disease ^(1), (2)^. Despite current intensive multimodality therapy, children diagnosed at an advanced stage have a dismal prognosis with an approximate 40% 5 year overall survival rate ^(2)^. Thus, to improve the prognosis of neuroblastoma patients with the intractable disease, new therapeutic strategies are required.

The most well-known genetic alterations in neuroblastoma are the amplification of the *MYCN* proto-oncogene and the gain of chromosome 17q. In addition, deletions of chromosomes 1p and 11q and hyperploidy are also commonly detected ^(4), (5), (6), (7)^. *MYCN* amplification as well as 1p and 11q deletions are significantly associated with poor prognosis for neuroblastoma patients, whereas hyperploidy is related to an excellent prognosis ^(6)^. Previously, anaplastic lymphoma kinase (*ALK*) was identified as an important oncogene implicated in familial and sporadic cases of neuroblastoma ^(8), (9), (10), (11)^. ALK was first identified as part of the nucleophosmin (*NPM*)*-ALK* fusion transcript derived from a t(2;5)(p23;q35) translocation, which was detected in anaplastic large cell lymphoma ^(12)^. ALK is a receptor tyrosine kinase that is widely expressed in the embryonic nervous system and it markedly declines after birth ^(13)^.

As more information is revealed regarding the molecular mechanisms underlying neuroblastoma pathogenesis as well as the genetic events leading to tumor initiation, maturation, and progression, there will be an increased understanding of the various clinical phenotypes, which will ultimately identify new therapeutic targets. In this review, we focus on the current advances in the genomic discoveries associated with neuroblastoma etiology.

## Neuroblastoma Clinical Presentation

**Figure 1.**
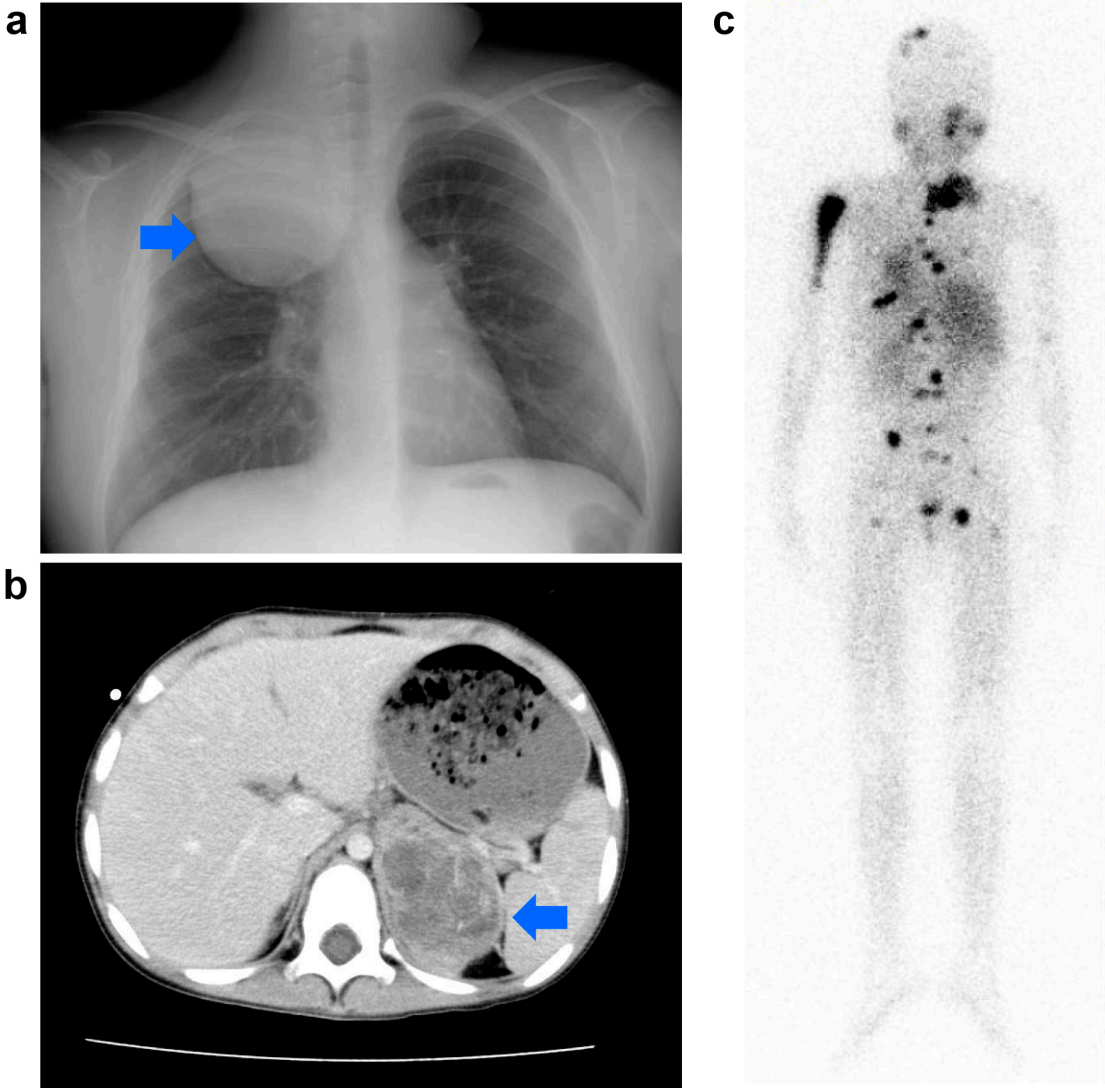
Clinical presentation of neuroblastoma. (a) Localized thoracic neuroblastoma. The arrow indicates tumor mass. (b) Enhanced abdominal computed tomography. The arrow indicates a large tumor in the adrenal medulla. (c), Metastatic neuroblastoma revealed by metaiodobenzylguanidine (MIBG) scintigraphy.

Neuroblastoma originates from neural crest progenitor cells and can arise anywhere in the sympathetic nervous system (Figure 1) ^(1), (2)^. Approximately 65% of primary tumors occur in the abdomen of which the majority arises in the adrenal medulla, whereas some develop in the paraspinal sympathetic ganglia ^(1), (2)^. Other common sites include the neck (5%), chest (20%), and pelvis (5%) ^(1), (2)^. The signs and symptoms of neuroblastoma are highly variable and depend upon age, primary tumor site, presence of metastatic disease, and occasionally on the associated paraneoplastic syndromes ^(1), (2)^. The international neuroblastoma staging system (INSS), established in 1989, is currently used to classify neuroblastoma patients into five stages. Stages 1 and 2 show distinguished localized tumors, stage 3 is characterized by an advanced locoregional disease, and stage 4 or 4s comprise metastatic tumors ^(14)^. More recently, a new presurgical international neuroblastoma risk group staging system (INRGSS) was proposed on the basis of clinical criteria and image-defined risk factors ^(15)^. INRGSS classifies neuroblastoma into L1 (localized disease without image-defined risk factors precluding surgical resection), L2 (localized disease with image-defined risk factors), M (metastatic tumors), and MS (metastatic tumors with metastases confined to the skin, liver, and bone marrow in children younger than 18 months of age). Neuroblastoma typically metastasizes to regional lymph nodes and bone marrow through the hematopoietic system ^(15)^. Neuroblastoma cells that have metastasized to the marrow are often involved in cortical bone. The tumor can also metastasize to the liver, most notably in cases with MS, and the involvement may be extensive; nevertheless, metastatic tumors often regress with no intervention other than supportive care ^(1), (15)^. The outcome of children with neuroblastoma depends on many factors, particularly, the INSS stage and age at diagnosis ^(1), (2)^.

Although improvements in outcome have been achieved with high-dose chemotherapy and the addition of 13-*cis*-retinoic acid, only one-third of children with high-risk cases are expected to be long-term survivors ^(16)^. More recently, it has been reported that monoclonal antibody-based therapies that specifically target disialoganglioside GD2 on neuroblastoma cells have improved response rates for high-risk neuroblastoma ^(17)^.

## Origin of Neuroblastoma

Approximately half of the neuroblastomas arise in the adrenal medulla (47%) followed by the abdominal/retroperitoneal (24%), thoracic (15%), pelvic (3%), and neck (3%) regions ^(1)^. Based on these common primary tumor sites and the biological features of neuroblastoma, it is widely accepted that the originating cell for neuroblastoma arises from neural crest-derived sympathoadrenal progenitor cells that differentiate to form sympathetic ganglion cells and adrenal chromaffin cells ^(18)^. The neural crest, originating from the embryonic ectoderm, develops from the neural tube after its closure and produces diverse cell types including peripheral neurons, enteric neurons and glia cells, melanocytes, Schwann cells, and cells of the craniofacial skeleton and adrenal medulla ^(18)^. During embryogenesis, neural crest cells subsequently undergo an epithelial-mesenchymal transition enabling the cells to delaminate, migrate, and differentiate into various cell types that contribute to the organism’s anatomical structures ^(18)^. This process is regulated by several mechanisms, such as a complex network of external signaling, activation of transcriptional programs, and epigenetic events. The dysregulation of the process of neural crest cell development can alter cell specification and deregulation of migration as well as cell differentiation, causing hyper-neoplastic lesions that may eventually result in neuroblastoma initiation and progression. Neuroblastoma derivers only from precursor cells or stem cells of the sympathoadrenal lineage but never from the other lineages derived from neural crest cells. Hence, the oncogenic events that cause neuroblastoma may occur after the point in which migrating cells choose to differentiate into sympathetic neurons.

**Figure 2.**
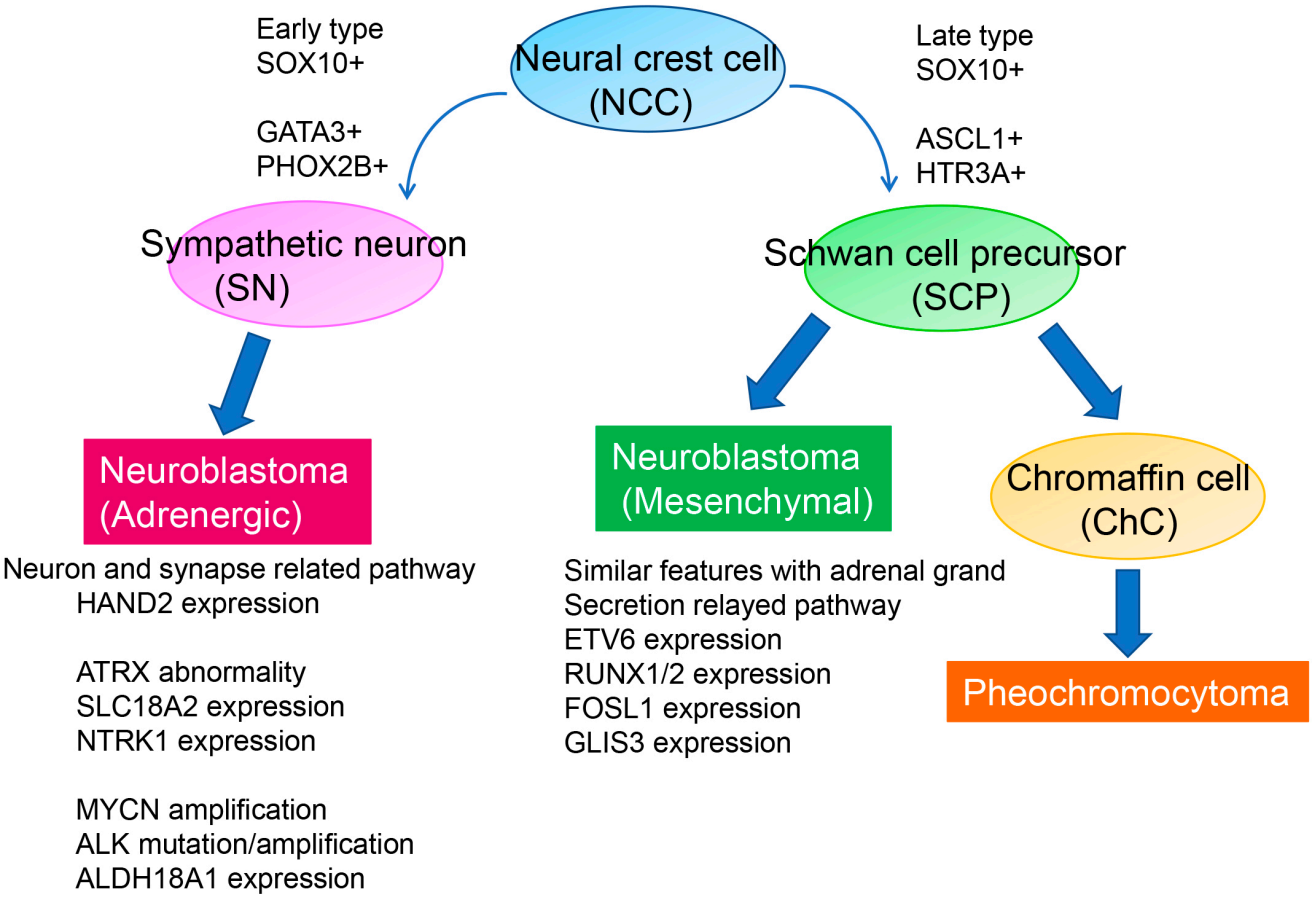
Summary of Noradrenergic (ADRN)-type and Mesenchymal (MES)-type neuroblastoma. (Cited and modified from van Groningen T, Koster J, Valentijn LJ, et al. Neuroblastoma is composed of two super-enhancer-associated differentiation states. Nat Genet. 2017;49(8):1261-6 ^(19)^. Copyright of the figure belongs to the authors.) Neuroblastoma was classified into Noradrenergic (ADRN)-type and Mesenchymal (MES)-type based on the superenhancer landscape of neuroblastoma. Commonly expressed genes during the course of neural crest differentiation and genetic abnormalities for each type are shown.

The superenhancer properties of neuroblastoma cell lines have revealed two neuroblastoma subtypes: a Noradrenergic (ADRN) type and a Mesenchymal (MES) type. These subtypes exhibit distinct expression patterns in core regulatory circuitry-related genes ^(19), (20)^. MES-type neuroblastoma cells and neural crest-derived precursor cells share common features, whereas ADRN-type cells are committed to the adrenergic lineage (Figure 2). Both cell types can spontaneously interconvert to generate neuroblastoma with high transcriptional plasticity.

## Chromosomal Copy Number Alterations

**Figure 3.**
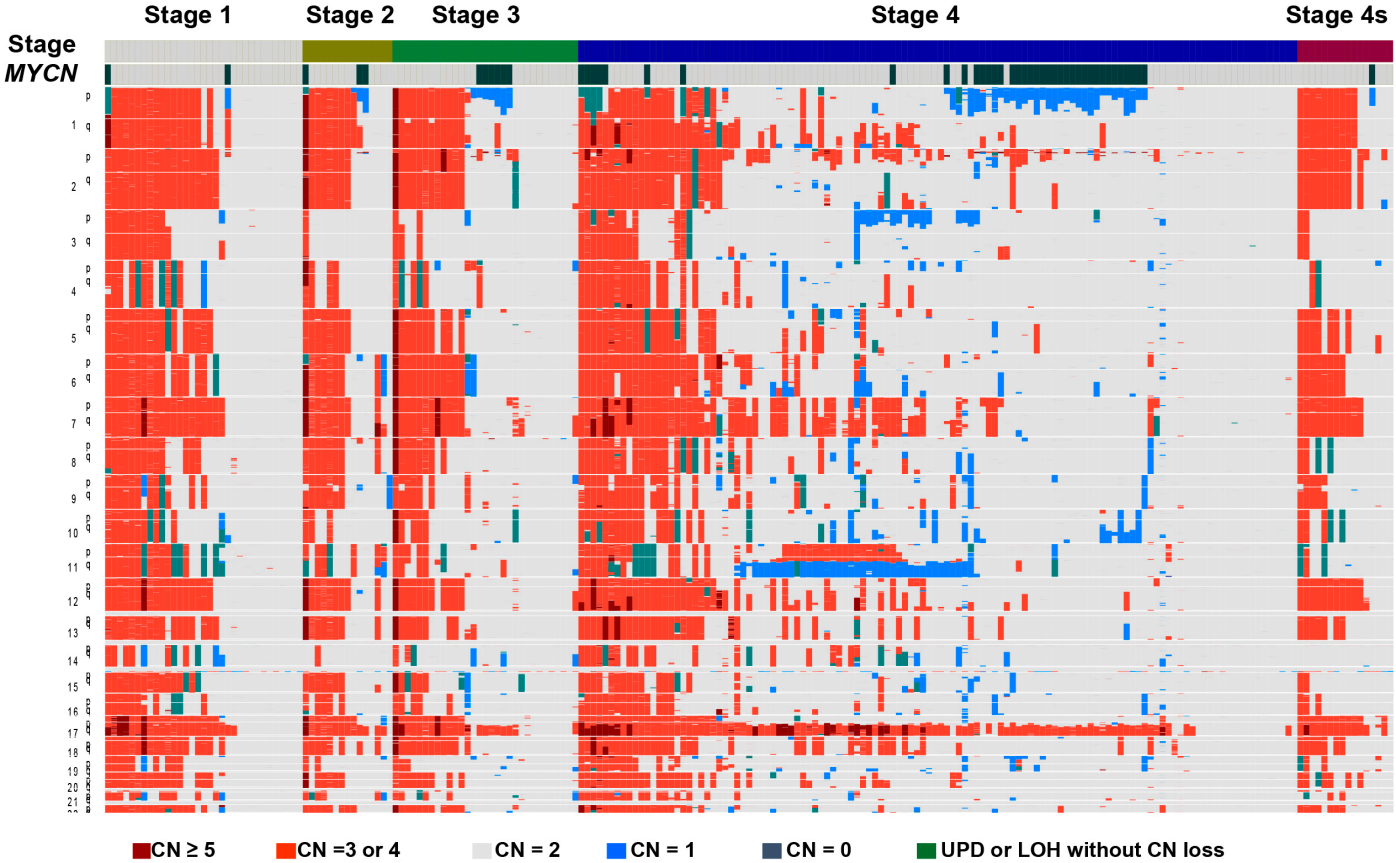
Copy number changes in primary neuroblastoma cases. Overview of copy number changes and allelic imbalances detected using a single nucleotide polymorphism array in 215 primary neuroblastoma cases. CN; copy number, UPD; uniparental disomy, LOH; loss of heterozygosity. Neuroblastoma patients were classified by the international neuroblastoma staging system (INSS).

Chromosomal copy number changes are the most common genetic event in neuroblastoma (Figure 3). The best-characterized copy number alteration associated with poor prognosis is the amplification of the *MYCN* oncogene ^(4)^. It was also reported that the loss of heterozygosity (LOH) on chromosome 1p correlates with poor prognosis of neuroblastoma and several candidate tumor suppressor genes have been identified in the common LOH regions of 1p including *TP73*, *CHD5*, *CAMTA1*, *KIF1B*, *CASZ1*, and *mir-34A* ^(21)^. However, because *MYCN* amplification and 1p LOH are not observed in approximately half of all high-risk neuroblastoma patients, it has been suggested that genetic aberrations other than *MYCN* amplification and 1p LOH are involved in the development and progression of the disease.

Chromosomal deletion of 11q can be detected in 35%-40% of primary neuroblastomas ^(6)^. Recently, several candidate genes responsible for 11q LOH, such as *CADM1* ^(22)^, *TSLC1* ^(23)^, *H2AFX* ^(24)^, and *ATM* ^(24)^, were reported by different groups. Because there is no mechanism ensuring their complete inactivation, our group and others revealed that synthetic lethality could be induced by treatment with a PARP inhibitor in neuroblastoma cells with *ATM* haploinsufficiency ^(24)^. Reportedly, ATM silencing promotes neuroblastoma progression independently of *MYCN* amplification ^(25)^. Notably, although the 11q deletion is predominantly detected in tumors without *MYCN* amplification and 1p LOH, it remains highly correlated with the dismal prognosis of neuroblastoma patients ^(6), (26)^. In a study comprising a large cohort of neuroblastoma cases registered with the Children’s Oncology Group study, 11q LOH and 1p LOH or *MYCN* amplification were independent poor prognostic markers as determined by multivariable analysis ^(6)^.

**Figure 4.**
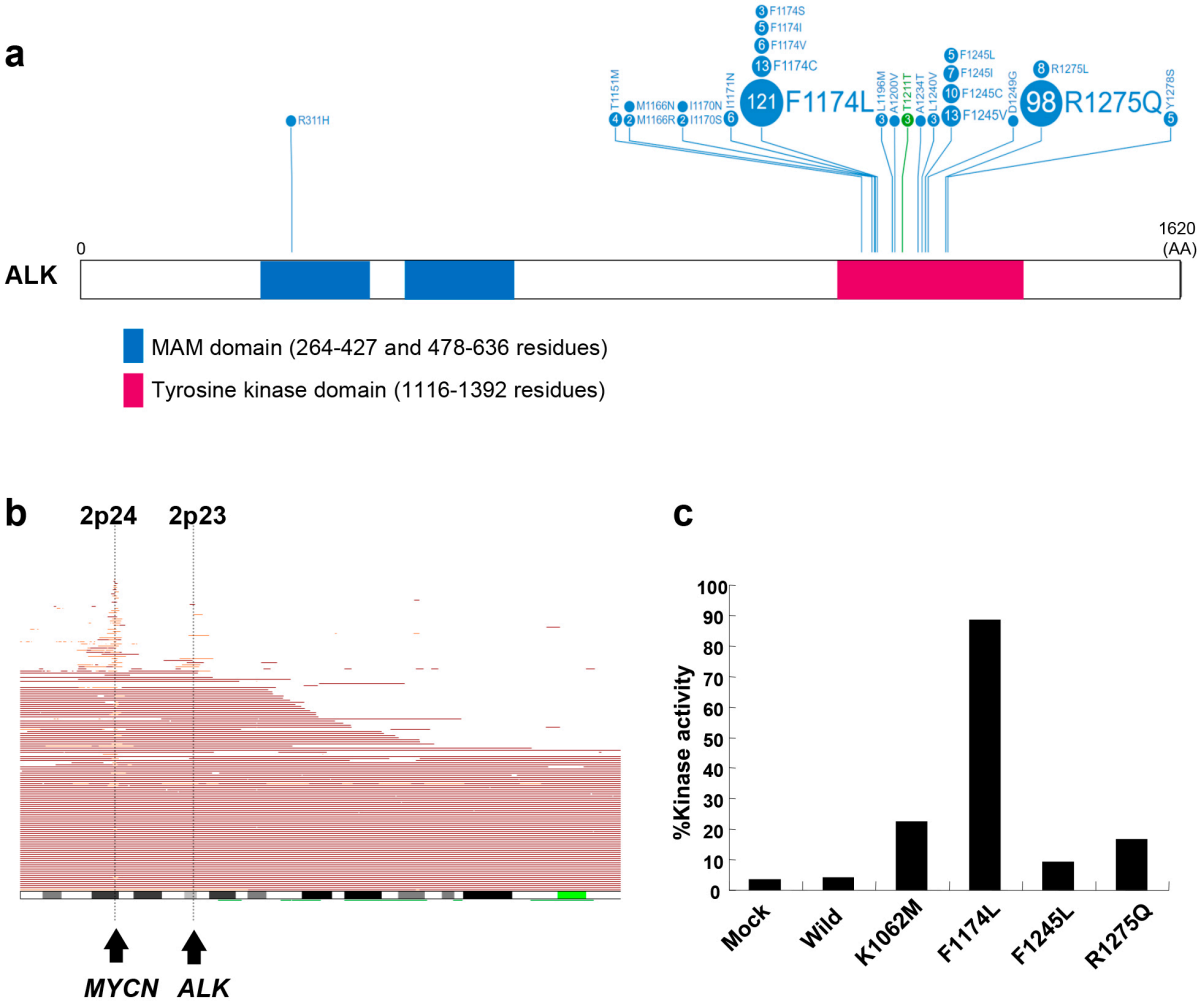
ALK mutations detected in neuroblastoma. (Cited and modified from Mosse YP, Laudenslager M, Longo L, et al. Identification of ALK as a major familial neuroblastoma predisposition gene. Nature. 2008;455(7215):930-5 ^(8)^ and Uryu K, Nishimura R, Kataoka K, et al. Identification of the genetic and clinical characteristics of neuroblastomas using genome-wide analysis. Oncotarget. 2017;8(64):107513-29 ^(26)^. Copyright of the figure belongs to the authors.) a. COSMIC frequencies of *ALK* mutations in neuroblastoma from the published literature with functional and therapeutic significance are shown. A blue circle indicates a missense mutation and a green circle indicates a silent mutation. The numbers in the circles represent the reported mutation numbers. The blue box indicates MAM domains and the pink box indicates the tyrosine kinase domain. b. Recurrent copy number (CN) gains in the 2p arm are defined by the *MYCN* locus on 2p24.3 and the *ALK* locus on 2p23, where vertical lines indicate common CN gains. High-grade amplifications are drawn in light red whereas simple gains are represented in dark red. c. Kinase activity of ALK mutants determined by *in* *vitro* kinase assays using the synthetic YFF peptide as a substrate.

*ALK* is an orphan receptor tyrosine kinase normally expressed only in the developing embryonic and neonatal central nervous system. Because of chromosomal translocation, ALK fusion proteins are constitutively active and have been characterized in various human malignancies ^(27)^. Previously, various genome-wide studies have revealed that *ALK* amplification and somatic mutations occur in <10% of primary neuroblastoma cases (Figure 4) ^(8), (9), (10), (11)^. Since ALK is located proximal to the *MYCN* locus, it can be coamplified with *MYCN;* however, solitary ALK amplification has rarely occurred. Additionally, mutations are found in almost all cases of familial neuroblastoma ^(8)^.

Besides the 17q gain, unbalanced translocations of 17q with 1p or 11q are found relatively frequent in neuroblastoma ^(7), (26)^. Previous studies have reported that high expression of *BIRC5*, *NME1*, *PPMID*, and *ncRAN* occurs in a subset of tumors with 17q gain, but the candidate genes responsible for this remain elusive ^(28), (29), (30), (31)^. Several other recurrent partial chromosomal imbalances have been detected by metaphase comparative genomic hybridization and single nucleotide polymorphism arrays including losses of 3p, 4p, 9p, and 19q and gains of 1q, 2p, 7q, and 11p ^(9), (26), (32)^. Furthermore, hemizygous deletions and sequence alterations of the chromatin remodeling genes, *ARID1A* (1p36) and *ARID1B* (6q25), were identified in a subset of cases and associated with early treatment failure and decreased survival ^(33)^. Subsequent functional analyses suggested that *ARID1A* and *ARID1B* were haploinsufficient tumor suppressor genes in *MYCN*-driven neuroblastoma ^(34)^.

## Chromosomal Rearrangements and Instability

Recent genomic studies of neuroblastoma tumors using whole-genome sequencing have identified loss-of-function genetic alterations (somatic mutations, small indels, and single nucleotide variations) of *ATRX*, which encodes chromatin remodeling proteins in the telomeric region, in approximately 10% of patients with neuroblastoma ^(35), (36)^. Patients whose tumors had *ATRX* alterations were typically older than 5 years, had an indolent disease course, and a dismal prognosis. Moreover, the rearrangements of the promoter region of *TERT* encoding the catalytic subunit of telomerase were detected in approximately 25% of neuroblastoma cases ^(35), (36)^. In support of an oncogenic role for *TERT*, neuroblastoma cell lines having rearrangements or *MYCN* amplification exhibited both upregulated *TERT* expression and enzymatic telomerase activity. *ATRX* and *TERT* alterations are involved in telomere maintenance through telomerase activity. Usually, they are not present in cases with *MYCN* amplification, suggesting that telomere lengthening represents a central mechanism that defines the high-risk group without *MYCN* amplification ^(37), (38)^.

Chromothripsis, a chromosomal instability phenomenon, describes a new oncogenic mechanism caused by many sudden rearrangements in the same cell in one or more chromosomes. This is in contrast to the conventionally described mechanism in which the accumulation of mutations over time causes cancer ^(39)^. Chromothripsis was observed in at least 2%-3% of all cancers, with the highest frequencies detected in soft tissue tumors ^(40)^. Recently, whole-genome sequencing also identified chromothripsis in advanced stages of neuroblastoma ^(41)^. Chromothripsis-related structural abnormalities are correlated with amplification of the *MYCN* or *CDK4* genes and 1p LOH ^(41)^, indicating that chromothripsis suppresses neuroblastoma cell differentiation through allelic loss of potential tumor suppressor genes on 1p36 involved in the induction of differentiation. Chromothripsis is also associated with chromosomal rearrangements of *TERT* resulting in a significant increase in telomere length ^(37), (38)^.

## Genetic Risk Factors

Genome-wide association studies (GWAS) further disclosed that neuroblastoma is a complex genetic disease related to common polymorphic alleles that can influence neuroblastoma development. At least 12 highly significant polymorphic alleles have been identified that can influence the development of neuroblastoma ^(42)^. Although each association has a modest individual effect on disease initiation, multiple perturbations can cooperate in an individual patient to promote malignant transformation during neurodevelopment. Many GWAS-defined neuroblastoma susceptibility genes have been identified including *CASC15*, *BRCA1*-associated RING domain protein 1 (*BARD1*), *LMO1*, *DUSP12*, *DDX4*, *IL31RA*, *HSD17B12*, *HACE1*, *LIN28B*, *LINC00340*, *LOC729177* (*FLJ44180*), and *NEFL*. These genes display oncogenic or tumor-suppressive functions related to the disease ^(43), (44)^. The discovery of these susceptibility loci demonstrates the utility of analyzing GWAS signals for clues into the underlying biology driving neuroblastoma genesis.

Conversely, unlike retinoblastoma, familial neuroblastoma is extremely rare (1%-2% of cases) ^(45)^. Familial neuroblastoma is typically consistent with an autosomal dominant pattern of inheritance with incomplete penetrance. A remarkable heterogeneity of clinical behavior is observed within pedigrees in terms of age at diagnosis, histology, and aggressiveness. Although familial neuroblastoma is very rare, these pedigrees provide a unique opportunity to identify the genetic drivers of neuroblastoma. The first predisposition gene identified in neuroblastoma was *PHOX2B*, a gene encoding a paired homeodomain transcription factor that promotes cell cycle exit and neuronal differentiation that plays a critical role in the development of neural crest-derived autonomic neurons. Germline mutations of *PHOX2B* occur in <10% of hereditary cases of neuroblastoma, whereas somatic *PHOX2B* mutations are rarely found in sporadic cases ^(46)^. *PHOX2B* mutations usually occur in neural crest-derived disorders, such as congenital central hypoventilation syndrome and Hirschsprung’s disease. The families with nonpolyalanine repeat expansion mutations typically experience the most severe phenotype, neuroblastoma-Hirschsprung’s disease-hypoventilation syndrome association. Thus, these observations suggest that perturbations in the PHOX2B-regulated differentiation pathway may be a common genetic factor responsible for these diseases derived from the neural crest. A more common lesion associated with familial neuroblastoma is found in the *ALK* locus. Approximately 80% of families with neuroblastoma harbor mutations in *ALK* and they have also been rarely found in germline and tumor cells of sporadic neuroblastoma ^(8), (9)^. Most of these mutations are located in the tyrosine kinase domain and lead to constitutive phosphorylation, indicating that *ALK* mutations found in neuroblastoma may be oncogenic drivers. Midkine and pleiotrophin are known as natural ligands of ALK ^(47)^. *ALK* expression in the developing sympathoadrenal progenitor of the neural crest is high and it may regulate the balance between proliferation and differentiation through multiple signal pathways including the MAPK and RAS-related protein 1 signal transduction pathways. Heritable mutations in *ALK* are the first example of a familial pediatric cancer arising from mutations in an oncogene.

## Spontaneous Regression

Spontaneous regression of neuroblastoma has been well documented in infants with stage 4S disease (e.g., patients <1 year of age with metastasis limited to the skin, liver, or bone marrow) ^(48)^. The strongest evidence for spontaneous regression arises from the mass screening studies undertaken in Japan, North America, and Europe ^(49), (50), (51)^. The actual prevalence of neuroblastoma regression is known; nevertheless, previous studies have provided evidence that regression may be at least as common as clinically detected neuroblastoma and probably about 200-fold higher than the clinically detected disease ^(48)^.

Segmental chromosomal aberrations have characterized the tumors from patients with stage 4 disease, whereas the majority of stage 4S tumors are near triploid with the whole chromosomal gains ^(52)^. Importantly, patients with 3p and 11q abnormalities in stage 4S tumors showed an inferior outcome compared with those without these alterations, particularly in *MYCN* single-copy tumors ^(53)^. The accurate mechanisms responsible for spontaneous regression are not fully known, but several plausible mechanisms have been proposed to date ^(54), (55), (56)^.

One of the candidate key mechanisms underlying tumor regression is the nerve growth factor (NGF) dependency of neuroblastoma cells ^(56)^. NGF binds to one of the neurotrophin receptors, TRKA, and high expression of *TRKA* has been observed in localized neuroblastoma and 4S tumors ^(57), (58)^. When cells derived from these tumors were cultured with endogenous NGF, they underwent neuronal differentiation and survived for months. By contrast, cell death by apoptosis can occur within a week in the absence of NGF ^(55)^. Hence, these in vitro culture conditions appear to recapitulate the behavior of *TRKA*-expressing neuroblastomas in patients with neuronal differentiation or spontaneous regression (apoptosis), depending on the presence or absence, respectively, of NGF in the microenvironment. Conversely, most high-risk neuroblastomas exhibited high telomerase activity and a poor prognosis, whereas the majority of the 4S tumors had low telomerase activity or short telomeres ^(59)^. These findings suggest that telomere crisis has a role in spontaneous tumor regression. Interestingly, when a neuroblastoma cell line was transfected with a dominant-negative form of telomerase, the cells displayed increased apoptosis ^(60)^. Furthermore, neuroblastoma cells with a dominant-negative form of telomerase exhibited reduced tumorigenicity in a mouse xenograft model. Thus, these data indicate that the loss of telomerase activity and telomere shortening are candidate mechanisms that lead to spontaneous regression of neuroblastoma.

Another potential explanation of spontaneous regression is tumor destruction mediated by an antitumor immune response. Approximately 50% of patients with paraneoplastic opsomyoclonus syndrome, which is correlated with antineuronal antibodies, differentiated tumors, and a favorable outcome in patients with neuroblastoma, present with neuroblastoma. This suggests that the other 50% either had neuroblastoma that regressed or a de novo autoimmune disease ^(61), (62), (63), (64)^. However, whether a humoral or cellular immune response mediates spontaneous regression remains unclear.

## Epigenetic Regulation

Epigenetic alterations affecting the expression of genes relevant to neuroblastoma development were initially reported over a decade ago and several studies have suggested that aberrations in gene DNA methylation or histone modification are related to clinical outcome ^(65), (66), (67), (68)^. Based on the analysis of promoter hypermethylation in 45 candidate genes in 10 neuroblastoma cell lines and 10 selected genes in 118 primary neuroblastomas through methylation-specific PCR, Alaminos et al. reported that the CpG island hypermethylation portrait showed distinct patterns for *MYCN*-amplified versus nonamplified tumors ^(69)^. They also discovered that promoter hypermethylation of the *HOXA9* was related to mortality in older patients compared with infants and tumors lacking *MYCN* amplification. By contrast, hypermethylation of the proapoptotic gene, *TMS1*, and the cell cycle-related gene, *CCND2*, was correlated with advanced-stage tumors ^(69)^. Additionally, it was reported that specific chromosomal regions could be identified as uniquely hypermethylated or hypomethylated in stage 4S tumors compared with other stages. They comprised transcription factors genes associated with neural crest development sympathetic neural differentiation ^(69)^. Notably, E2F1 binds to the *TERT* promoter which is hypermethylated in stage 4S compared with stage 4 tumors. Lower expression of *TERT* was observed in stage 4S compared with stage 4 tumors ^(69)^ indicating that *TERT* DNA methylation also regulates telomerase activity in stage 4S neuroblastoma.

More recently, genome-wide methylation analysis using Infinium Human 450 K BeadChips resulted in more comprehensive studies. Henrich et al. applied an integrative approach to analyze the global methylation patterns, transcriptomes, and copy number aberrations of 105 neuroblastoma cases, complemented by primary tumor- and cell line-derived global histone modification analyses and epigenetic drug treatment in vitro ^(70)^. This study further revealed the presence of distinct DNA methylation patterns that identified distinct subgroups which correlated with survival and clinical/biological variables, including *MYCN* amplification and copy number changes ^(70)^. Similar results were reported by Gomez et al. on the basis of the same approach using 35 primary neuroblastomas ^(71)^. In addition, non-CpG methylation was observed and mostly associated with tumors characterized by favorable clinicopathological features.

## Immunology and Immunotherapy

Initial evidence suggesting the existence of an immune response to neuroblastoma was provided in 1968 when blood leukocytes from neuroblastoma patients, including 50%-70% lymphocytes, inhibited colony formation and exhibited cytotoxicity against their neuroblastoma cells and allogeneic neuroblastoma cells ^(72)^ *in vitro*. Also, tumors from infant cases contained high numbers of leukocytes ^(69), (73)^ and neuroblastoma in infants often showed spontaneous regression ^(74), (75)^. Together, these findings suggest that neuroblastoma has a characteristic immune mechanism and the development of an antineuroblastoma therapy based on the immune system is warranted.

Several studies support the importance of T cells and natural killer (NK) cells in the immune response to cancer, including neuroblastoma ^(76), (77)^. Normally, cytotoxic T cells (CTLs) exhibit cytotoxic activity upon presentation of HLA class 1, but most neuroblastoma cells do not express HLA class I and II molecules and thus could represent better targets for NK cells than for CTLs ^(77)^. Recent studies revealed an antitumor role for NK cells in high-risk neuroblastoma patients. Venstrom et al. reported that killer immunoglobulin-like receptor (KIR) and HLA gene polymorphisms interact to govern NK cell function associated with disease progression and survival in high-risk neuroblastoma cases treated with autologous hematopoietic stem cell transplant (AHSCT) ^(78)^. Those with a “missing ligand” KIR-HLA compound genotype had a 46% lower risk of death at 3 years after AHSCT compared with patients who possessed all ligands for inhibitory KIR ^(78)^. Among all KIR-HLA combinations, 16 patients lacking the HLA-C1 ligand for KIR2DL2/KIR2DL3 exhibited the highest 3 year survival rate (81%). In this study, the survival rate was more strongly associated with a “missing ligand” than with tumor *MYCN* amplification ^(78)^. Thus, NK cells have a promising role in immunotherapy in high-risk neuroblastoma ^(79)^.

The most obvious contribution of monoclonal antibodies (mAbs) to high-risk neuroblastoma treatment came from the discovery of a high-level expression of disialoganglioside (GD2) in neuroblastoma cells and from the generation of mAbs to this surface molecule ^(17)^. A phase I/IB study assessed the combination of IL-2 and murine anti-GD2 antibody 14G2A in patients with recurrent neuroblastoma ^(80)^. Phase I studies were also done to evaluate the chimeric anti-GD2 mAb ch14.18 in refractory or relapsed patients ^(81)^ and in patients who responded to high-dose therapy and AHSCT ^(82)^. A subsequent phase I study tested the combination of antibody-dependent cellular cytotoxicity-enhancing cytokines (GM-CSF and IL-2) and ch14.18 therapy combined with cis retinoic acid (RA) following high-dose therapy and AHSCT and found the regimen to be tolerable ^(83)^. This clinical trial progression culminated in a recently completed phase III randomized study of cisRA together with ch14.18, IL-2, and GM-CSF vs. cisRA only for patients with high-risk neuroblastoma who had a clinical response to induction therapy and myeloablative consolidation therapy/AHSCT. Immunotherapy after consolidation significantly improved event-free survival and overall survival ^(17)^. Hence, these findings indicate that anti-GD2 therapy combined with GM-CSF and IL-2 will improve the survival of patients with high-risk neuroblastoma.

Other immunologic approaches for high-risk patients with neuroblastoma include the development of chimeric antigen receptor-modified T cells (CAR-T) and Bi-specific T-cell Engager ^(84), (85)^. Although these approaches primarily use GD2 as a tumor antigen at present, their clinical effectiveness remains unknown.

## Conclusion

Biological findings facilitate our understanding of neuroblastoma heterogeneity and improve disease management and prognosis. This review summarizes our understanding of neuroblastoma pathogenesis and the promising therapeutic strategies. The overall survival rate in cases with neuroblastoma is gradually improving because of the introduction of multidisciplinary treatments. Nevertheless, the prognosis for high-risk neuroblastoma remains poor and continued development of innovative therapies that include new drugs, novel immunotherapies, and other strategies is required.

## Article Information

### Conflicts of Interest

None

### Sources of Funding

This work was supported by JSPS KAKENHI grant numbers JP19J11112, JP17H04224, JP18K19467, and JP20H00528; Project for Cancer Research and Therapeutic Evolution P-CREATE grant number JP19cm0106509h9904 and Practical Research for Innovative Cancer Control grant number JP19ck0106468h0001 from Japan Agency for Medical Research and Development (AMED); and Princess Takamatsu Cancer Research Fund.

### Acknowledgement

I am grateful to Ms. Matsumura, Ms. Hoshino, Ms. Yin, Ms. Saito, Ms. Mori, Ms. Mizota, and Ms. Nakamura for their excellent technical assistance. I also wish to express my appreciation to Dr. Seishi Ogawa and Dr. Hiroo Ueno, Kyoto University, Dr. Yasuhide Hayashi, Jyobu University, and Dr. Yuyan Chen, The University of Sydney for their continuing support.

### Approval by Institutional Review Board (IRB)

All study procedures were approved by the Research Ethics Committee of the University of Tokyo (permit number: G1598) and Kyoto University (permit number: G1030-8).
